# Active piezoelectric bone conduction implant Osia^®^ 2 - evaluation of surgery and one-year audiological and quality of life benefits

**DOI:** 10.1007/s00405-024-09057-2

**Published:** 2024-11-09

**Authors:** Wojciech Gawęcki, Renata Gibasiewicz, Magdalena Błaszczyk, Małgorzata Wierzbicka, Ewelina Bartkowiak

**Affiliations:** 1 Department of Otolaryngology and Laryngological Oncology, Poznan University of Medical Sciences, Poznań, Poland; 2 Medicus sp. z o.o, Wrocław, Poland; 3 University of Silesia in Katowice, Katowice, Poland; 4 Faculty of Medicine, Wroclaw University of Science and Technology, Wrocław, Poland; 5 Institute of Human Genetics, Polish Academy of Sciences, Poznań, Poland

**Keywords:** bone conduction, piezoelectric actuator, active hearing implant, Osia^®^

## Abstract

**Purpose:**

To present the surgical outcomes and one-year audiological and quality of life results of implantation of the Osia^® ^2 active piezoelectric bone conduction implant.

**Methods:**

Twenty adults with mixed and conductive hearing loss were implanted with the Osia^®^ 2 system and followed up for at least one year. The surgical course, healing, and soft tissue condition were assessed. Audiometric tests included pure tone audiometry, speech audiometry and direct bone conduction measurements. Participants completed the APHAB and SSQ questionnaires.

**Results:**

All surgeries were successful. No soft tissue problems were observed. Pure tone audiometry showed a mean functional gain of 47.4 ± 5.6 dB HL (*p* = 0.000089). The Polish Monosyllabic Word Test showed mean improvements for silent, medium and loud speech of 59.5 ± 1.8%, 46.5 ± 32.3% and 13.3 ± 20.9% in quiet and 38.5 ± 24.4%, 62.0 ± 9.1%, and 36.5 ± 34.4% in noise (all *p* < 0.05). The Polish Matrix Test indicated a mean SNR improvement of 8.78 ± 2.31 dB SPL (*p* = 0.000155). BC in situ measurements were significantly better post-implantation compared to preoperative levels with Baha^® ^6 Max on the Softband. APHAB scores showed significant improvements in global, ease of communication, background noise and reverberation scores (all *p* < 0.001). SSQ scores improved significantly in speech, spatial and quality subscales (all *p* < 0.000001).

**Conclusion:**

The Osia^® ^2 implantation is an effective treatment for patients with mixed and conductive hearing loss. The surgery is relatively easy and safe, with no significant postoperative or magnet pressure-related complications. Osia^® ^2 significantly improves speech understanding in noise and reduces communication problems.

## Introduction

Contemporary medicine, thanks to modern technology, offers many treatment options for hearing-impaired patients: tympanoplastic surgery, hearing aids and various implantable systems. Nowadays, four types of hearing implants are available on the market: cochlear implants, bone conduction implants, middle ear implants and auditory brainstem implants [1–4].

Bone conduction implants (BCIs), also known as bone-anchored hearing aids (BAHAs), are well-established solutions for treating patients with unilateral and bilateral, mixed and conductive hearing loss, as well as those with single-sided deafness [5–7]. Since the first implantation described by Tjellström and Granström in 1977 [8], many different systems have been developed, which can be generally divided into passive and active systems [2, 7, 9].

In passive bone conduction systems, the external sound processor with the transducer (vibrator) is located outside the body, exactly behind the ear, and the vibrations produced by the transducer have to be transmitted to the implanted part of the system, which is located inside the temporal bone. Depending on the method of connection between the processor and the implant, a distinction can be made between the percutaneous passive devices (with percutaneous abutment) and transcutaneous passive devices (with a system of magnets). Both types of these passive systems have been in use for many years and both have certain drawbacks and limitations [6, 9]. Percutaneous passive BCIs interrupt the continuity of the skin barrier and therefore require lifelong and daily hygiene and pose a risk of local skin complications [10]. Furthermore, the cosmetic effect is not optimal and some patients who could benefit from the system opt out [11]. On the other hand, in transcutaneous passive BCIs, sound quality can be limited due to sound attenuation caused by the skin between the magnets [11–13]. Moreover, constant pressure on the skin can lead to redness or pain over the magnet [14] and sometimes even soft tissue necrosis [15].

In active bone conduction systems, only the external sound processor is located outside the body (posteriorly and upwards from the auricle), but the transducer is located directly in/on the patient’s bone. The processor is attached to the head and connected to the implanted part of the system by magnetic attraction force (active transcutaneous system) [6, 9, 16]. This concept was designed to address problems related to passive devices, such as skin discontinuity, poor aesthetic effect, soft tissue attenuation and skin pressure. Depending on the type of transducer, active systems can be divided into electromagnetic and piezoelectric. The electromagnetic transducer contains a magnet, coil and counterweight. In such a vibrator, an alternating polarity signal from the sound processor causes an electromagnetic field production that excites the magnet element and counterweight. The first electromagnetic active device, Bonebridge^®^ (Medel, Austria), was launched in 2012 [16], and another system, Sentio (Oticon, Denmark), appeared on the market a few months ago. The only piezoelectric bone conduction system currently available is Osia^®^ (Cochlear Ltd, Australia). In a piezoelectric vibrator, a crystalline material is formed into compact wafers that are laminated or bonded together. The electrical stimulation of this material causes contraction and expansion that follows the electrical signal. The use of a piezoelectric transducer has a number of advantages, namely a reduction in MRI constraints, stronger amplification, smaller size and simpler design of the transducer, which in turn translates into a simplified surgical technique and lower failure rate. The Osia^®^ received CE in 2019 and the device was available, as an early market release, for 8 selected European clinics. The safety and efficiency of this device has been confirmed in many studies [17–24]. To simplify the surgical procedure, the device was modified and marketed as the Osia^® ^2 system, first in the USA (received FDA in 2019) and then in Europe (received CE in 2021). The results of a Controlled Market Release study conducted in the United States between December 2019 and February 2020 and preliminary outcomes from a single centre were published by Goldstein et al. and confirmed the safety of the surgery and excellent audiological results [6]. The Osia^® ^2 system has been available in our country and in our department since November 2021.

The aim of this study was to present the surgical outcomes and one-year audiological and quality of life results of implantation of this active piezoelectric Osia^®^ 2 system.

## Materials and methods

### Study design and patients characteristics

The prospective study was conducted between November 2021 and March 2024 in a tertiary referral clinical centre. Twenty adult patients with unilateral and bilateral mixed and conductive hearing loss, candidates for bone conduction device implantation, were enrolled and implanted with the Osia^®^ 2 system between November 2021 and March 2023 and followed up for at least one year. The details of patients are presented in Table 1 and the study protocol is shown in Table 2.

**Table 1:** Patients’ characteristics

Patients	20 patients; Male – 7, Female – 13
Age (years)	Adults; Mean: 50 (min 24, max 73)
Hearing loss (HL)	16 x bilateral HL (10 x bilateral mixed HL, 4 x mixed & sensorineural HL, 1 x conductive & mixed HL,1 x conductive & sensorineural HL) 4 x unilateral HL (3 conductive, 1 mixed)
Etiology of HL	14 x chronic otitis media – after surgical treatment (12 after CWD mastoidectomy, 1 after CWU mastoidectomy, 1 after myringoplasty) 3 x otosclerosis – after unsuccessful stapedotomy 3 x atresia of external auditory canal
Pure Tone Average (PTA4: 0.5,1,2,4 kHz)	Air Conduction – mean 71 dB HL (min 46, max 95) Bone Conduction – mean 31 dB HL (min 13, max 48)
Side of implantation	11 x right and 9 x left

**Table 2:** The study protocol

Visit	Time	Definition
V1	qualification	unaided and Baha^®^ 6 Max audiological tests, QoL questionnaires
V2	0 d	surgery
V3	7–10 d	control visit after surgery
V4	1 mth	SP fitting, Osia^®^ 2 audiological tests
V5	6 mths	control visit, Osia^®^ 2 audiological tests, QoL questionnaires
V6	12 mths	control visit, Osia^®^ 2 audiological tests, QoL questionnaires

### Osia^®^ 2 system

The Osia^®^ 2 system evaluated in this study is composed of two implantable parts (BI300 bone conduction implant and OSI200 active piezoelectric implant) and an external sound processor. The audiological indications for the system are unilateral and bilateral, mixed and conductive hearing loss, as well as single-sided deafness. In cases with mixed hearing loss, the bone conduction pure tone average in the implanted ear must not exceed 55 dB HL [25].

### Surgery and fitting

**Fig. 1:**
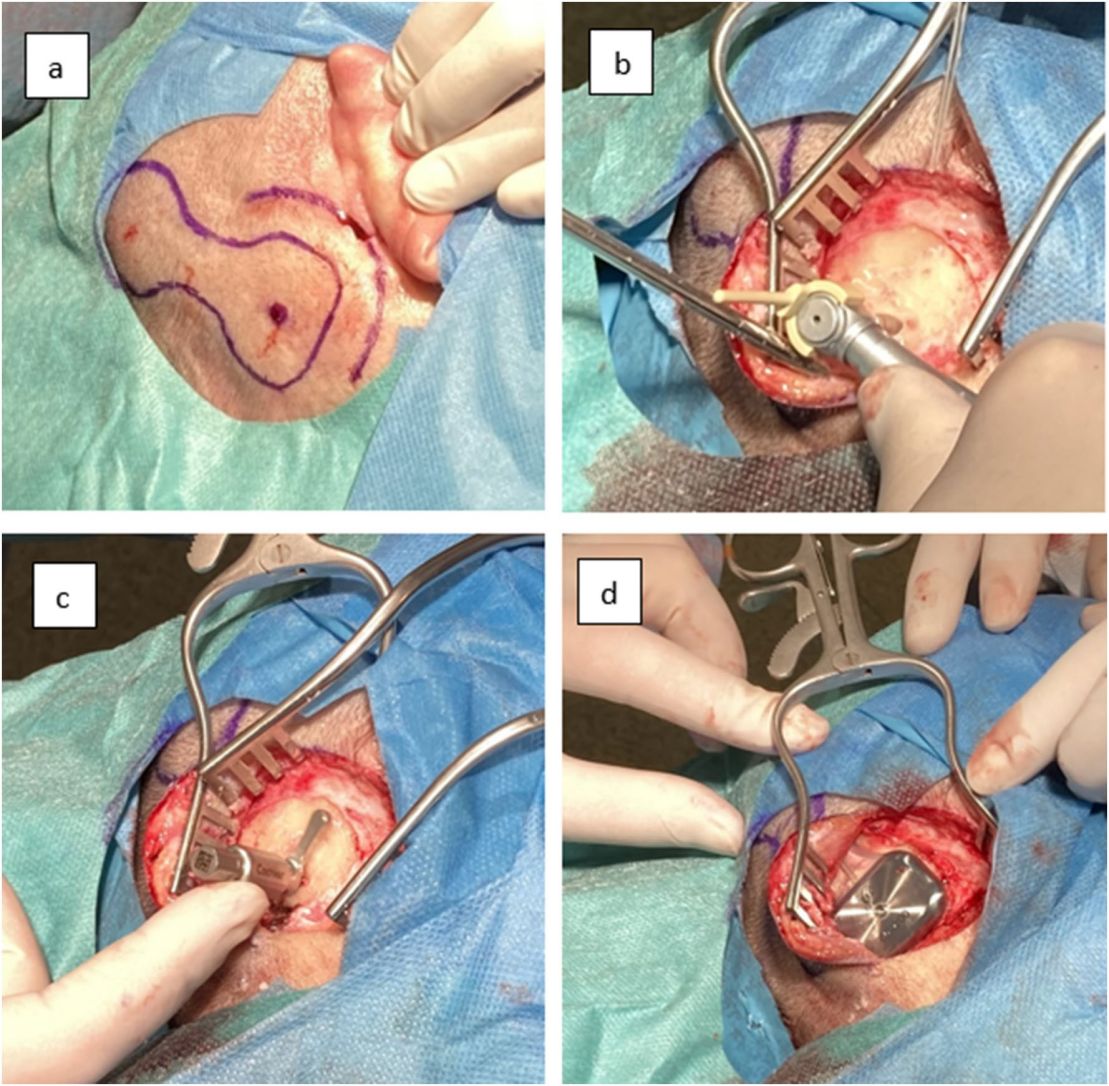
Surgery of Osia^®^ 2 (right side): (**a**) the place for the device and incision line marked, (**b**) drilling a hole for BI300 implant, (**c**) implant BI300 in place, subperiosteal pocket prepared, bone around reduced, bone surface checking, (**d**) the whole device in place (OSI200 connected to BI300 implant)

All surgeries were performed under general anaesthesia by a single experienced otosurgeon (WG). The surgical technique was as described in the Cochlear surgical guide [26]. In all cases, the skin incision was slightly modified, creating a C-shaped incision forward and downward from the planned implant side (Fig. 1). All cases received three doses of antibiotic at 8-hour intervals (the first dose was given just before surgery). The sound processor was fitted one month after surgery.

### Evaluated parameters

The following parameters, divided into three groups, were evaluated:

1. Surgery, healing and state of soft tissue in the operated area: surgery time, implant position, soft tissue reduction, bone polishing, any problems or difficulties during surgery, healing process, pain and numbness, state of soft tissue during one-year follow-up.
2. Audiological tests:
  - pure tone audiometry performed (1) in headphones (air and bone conduction) and (2) in the free field: first, before surgery unaided and with Baha^®^ 6 Max sound processor on the Softband, and then after surgery with the implanted Osia^®^ 2 system; free field thresholds were measured using warble tones presented from a loudspeaker that was situated 1 m in front of the patient,
  - speech audiometry with the Polish Monosyllabic Word Test performed (1) in headphones and (2) in the free-field, both in quiet and in noise (the speech of 50 dB, 65 dB and 80 dB SPL was presented from the loudspeaker that was situated 1 m in front of the patient; the noise of 55 dB SPL was presented from the loudspeaker placed behind the patient); first before surgery, unaided and with the Baha^®^ 6 Max sound processor on the Softband, and then after surgery with the implanted Osia^®^ 2 system,
  - speech audiometry with the Polish Sentence Matrix Test (adaptive sentence test in noise) performed in the free-field (the speech was presented from the loudspeaker that was situated 1 m in front of the patient; the noise was presented from the loudspeaker placed behind the patient); first before surgery, unaided, and then after surgery with the implanted Osia^®^ 2 system,
  - direct bone conduction (BC in situ) through the Baha^®^ 6 Max sound processor on the Softband before surgery and through the implanted Osia^®^ 2 device after surgery.
3. Quality of life benefits evaluated by comparing preoperative and postoperative results of APHAB (Abbreviated Profile of Hearing Aid Benefit) and SSQ (Speech, Spatial and Qualities of Hearing Scale) questionnaires.

Audiological evaluation was performed before surgery and 1, 6 and 12 months after surgery. All audiological tests were performed with an Otometrics Madsen Astera device in a soundproof room. During all free field tests, the contralateral ear was blocked with an earplug. The quality of life evaluation was performed before surgery and 6 and 12 months after surgery.

The investigation was approved by the local Ethics Committee [decision number 770/21].

### Statistical analysis

Statistical analysis was performed according to a predefined study design: all patients were treated with surgery and the efficacy study consisted of comparing outcomes before surgery and follow-up visits. All statistical analyses were paired and non-parametric. The Wilcoxon-signed rank test was used for all analyses. All tests were two-sided and performed at the 0.05 significance level (α = 0.05).

## Results

### Surgery, healing, state of soft tissue

In all cases, the surgery was successful, without any significant problems or complications. The average surgery time was 64.5 min, ranging from 51 to 91 min depending on the anatomical conditions. The position of the BI300 was as planned in 13 cases, but in 6 it was altered 5–8 mm upwards due to the position of the emissary vein or unfavourable bone curvature/surface, and in 1 it was altered posteriorly to avoid implantation into the bone suture. As the thickness of the soft tissue in the coil region in our patients ranged between 4 and 8 mm, no soft tissue reduction was required in any case. Bone polishing was performed in 14 cases – in 8 moderately and in 6 significantly. During one surgery, a defective Vibrant Soundbridge^®^ implant (MED-EL) was removed prior to Osia^®^ 2 implantation. The pictures from the Osia^®^ 2 surgery are presented in Fig. 1.

In two cases, a small hematoma was detected the day after surgery (during dressing changes), which was removed by compression and suction and did not require any additional surgery. These cases were treated with a pressure dressing and received an antibiotic for 7 days. Two other patients complained of temporary pain, which disappeared after a few weeks. Besides, there were no problems with healing. Some patients experienced mild pain up to a few days after surgery and a slight decrease in skin sensitivity up to a few weeks after surgery, but after six and twelve months there was no pain or numbness in the operated area. During one-year follow-up, no soft tissue problems were observed, including those caused by the pressure of the magnet.

### Audiological benefits

#### Pure tone audiometry

**Fig. 2:**
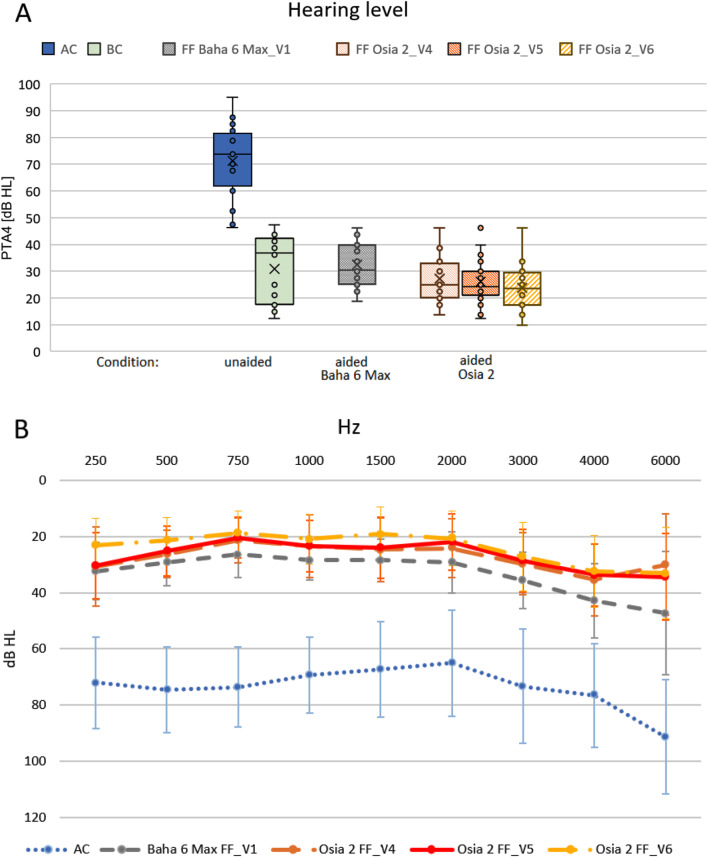
The results of pure tone audiometry in the free field with the implanted Osia^®^ 2 device in comparison to preoperative results - unaided and with Baha^®^ 6 Max processor on the Softband: (**A**) - box plot of the distribution of PTA4 hearing thresholds, (**B**) – results for individual frequencies (AC – air conduction, BC – bone conduction); V1 – before surgery, V4 – one month after surgery, V5 – six months after surgery, V6 – one year after surgery

A significant improvement in pure tone audiometry in the free field was observed with the implanted Osia^®^ 2 device in comparison to unaided hearing. One year after surgery, the mean functional gain (comparison of hearing with the device in the free field to air conduction) was 47.4 ± 5.6 dB HL (*p* = 0.000089). The obtained results were 8.6 dB HL better than those for Baha^®^ 6 Max processor on the Softband (38.8 ± 6.2 dB HL, *p* = 0.000089). The details are presented in Fig. 2.

#### Speech audiometry with the Polish Monosyllabic Word Test

**Fig. 3:**
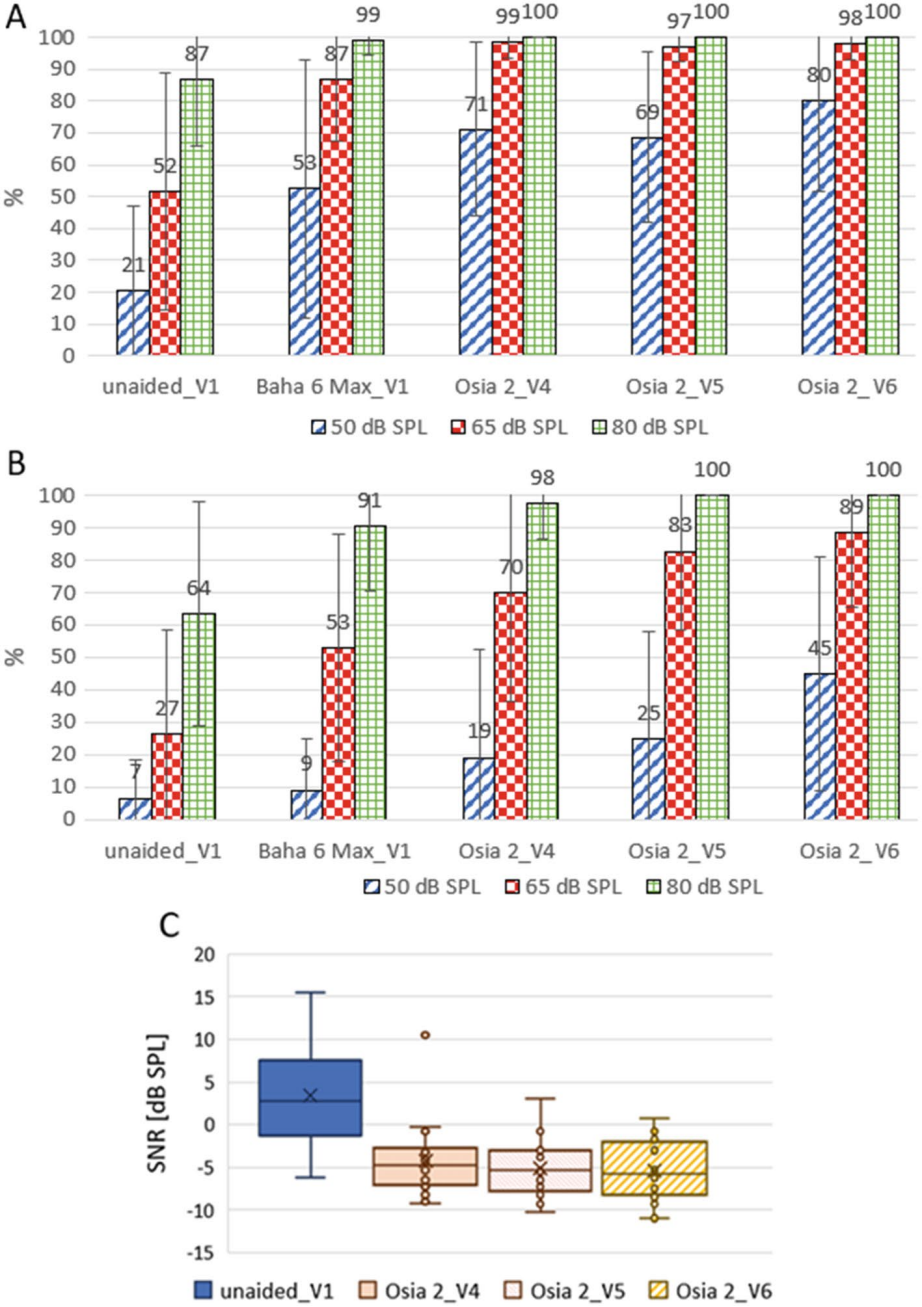
The results of speech audiometry: (**A**) - with Polish Monosyllabic Word Test in quiet, (**B**) - with Polish Monosyllabic Word Test in noise, (**C**) - with Polish Sentence Matrix Test; V1 – before surgery, V4 – one month after surgery, V5 – six months after surgery, V6 – one year after surgery

A significant improvement in speech understanding in the free field in both quiet and noise was observed with the implanted Osia^®^ 2 device compared to the unaided situation. For silent, medium and loud speech (50 dB, 65 dB and 80 dB), the mean improvement one year after surgery in comparison to the unaided situation was 59.5 ± 1.8% (*p* = 0.000151), 46.5 ± 32.3% (*p* = 0.000196) and 13.3 ± 20.9% (*p* = 0.017961) for speech in quiet, and 38.5 ± 24.4% (*p* = 0.002093), 62.0 ± 9.1% (*p* = 0.000189) and 36.5 ± 34.4% (*p* = 0.000982) for speech in noise. Generally, the results after the Osia^®^ 2 surgery were also better compared to those with the Baha^®^ 6 Max processor on the Softband, especially for silent speech in quiet and in noise and for medium speech in noise. The details are demonstrated in Fig. 3.

#### Speech audiometry with the Polish Sentence Matrix Test

The results of this adaptive test in noise showed an improvement of the mean SNR of 8.78 ± 2.31 dB SPL (*p* = 0.000155) one year after Osia^®^ 2 surgery compared to unaided hearing (mean SNR before implantation in the unaided condition was + 3.43 dB SPL and one year after surgery with Osia^®^ 2 device was − 5.35 dB SPL). The details are presented in Fig. 3.

#### Direct bone conduction

**Fig. 4:**
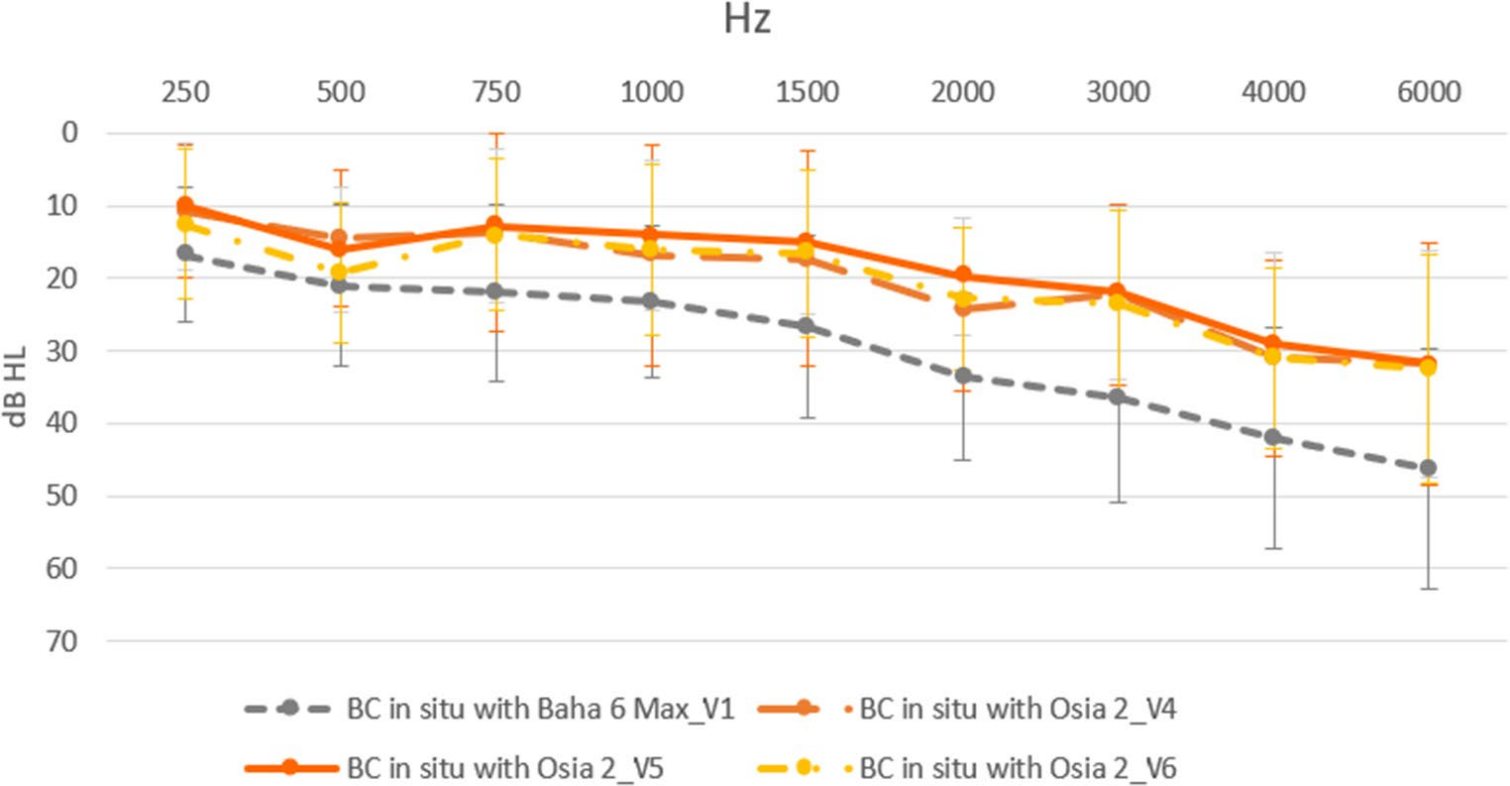
The difference between bone conduction in situ measurements with the implanted Osia^®^ 2 device and those performed preoperatively with Baha^®^ 6 Max on the Softband; V1 – before surgery, V4 – one month after surgery, V5 – six months after surgery, V6 – one year after surgery

For all frequencies, the results of the bone conduction (BC) in situ measurements with the implanted Osia^®^ 2 device were better in comparison to the preoperative levels with Baha^®^ 6 Max on the Softband. One year after surgery, the mean BC in situ (for 0.5, 1, 2 and 4 kHz) improved by 7.69 ± 2.54 dB (*p* < 0.000001). However, the biggest improvement was observed for 6 kHz, which amounted to 13.75 ± 0.78 (*p* = 0.000629). The details are presented in Fig. 4.

### Quality of life results

**Fig. 5:**
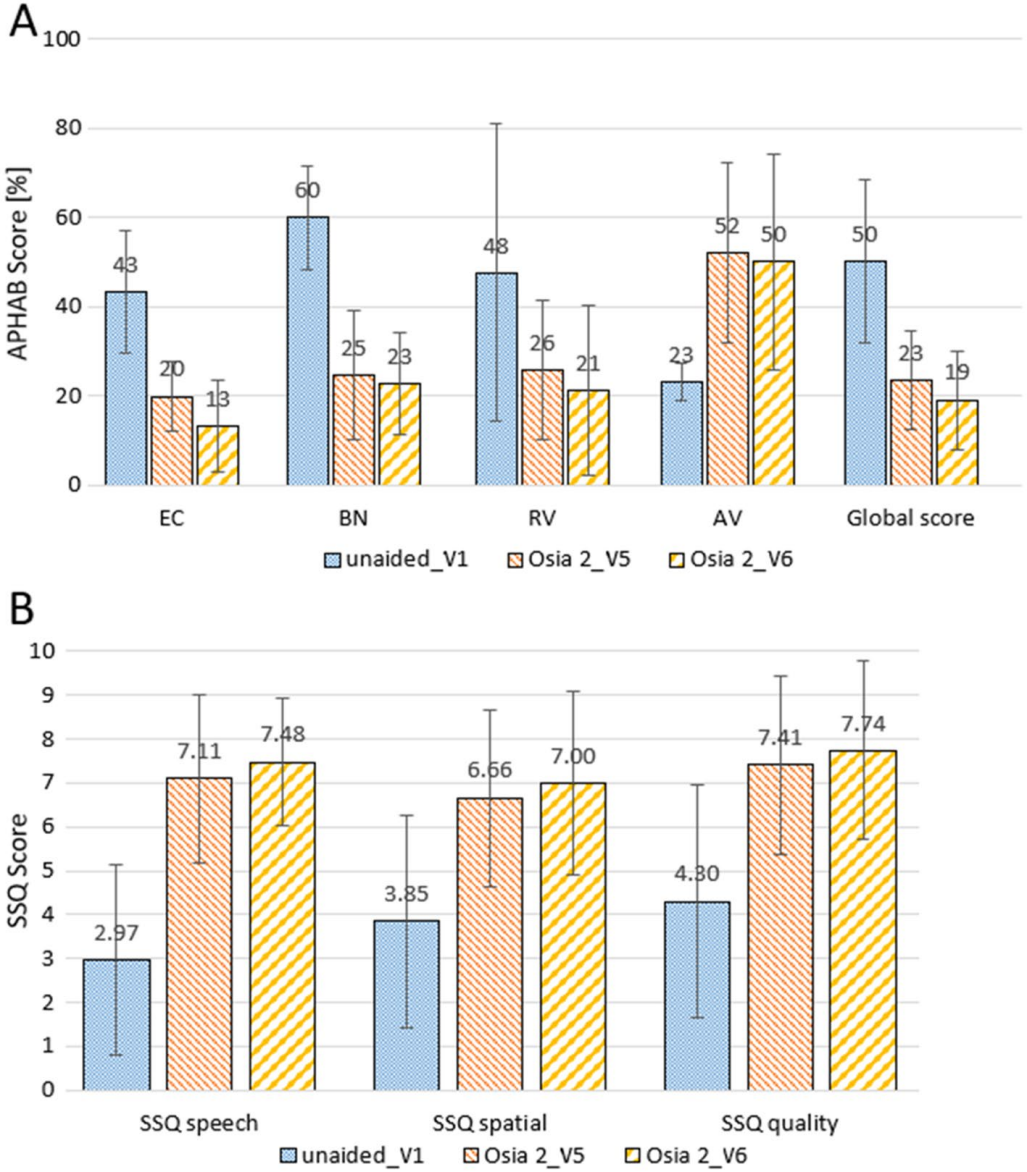
The benefits from Osia^®^ 2 implantation evaluated by Quality of Life Questionnaires: (**A**) - APHAB scale (EC – ease of communication, BN – background noise, RV – reverberation, AV – aversiveness, Global score – mean of EC, BN and RV), (**B**) - SSQ scale; V1 – before surgery, V5 – six months after surgery, V6 – one year after surgery

The number of hearing problems evaluated with the APHAB scale in different acoustical situations was significantly reduced after implantation in global score and all subscales except aversiveness. One year after surgery, the mean improvement in global score, ease of communication score, background noise score and reverberation score was 31.1 ± 8.5% (*p* = 0.000089), 30.0 ± 15.9% (*p* = 0.000254), 37.1 ± 6.2% (*p* = 0.000089) and 26.3 ± 6.7% (*p* = 0.000449), respectively. Similarly, after the implantation of the Osia^®^ 2 system, an evident improvement was observed in speech, spatial and quality of hearing measured by the SSQ scale. One year after surgery, the SSQ score improved by 4.51 ± 0.73 for speech (*p* < 0.000001), 3.15 ± 0.32 for spatial (*p* < 0.000001) and 3.44 ± 0.62 for quality subscale (*p* < 0.000001). The details are presented in Fig. 5.

## Discussion

In this study, the surgery and one-year audiological and quality of life results of the active piezoelectric bone conduction system Osia^®^ 2 are presented. The results of our study showed the safety of surgery and an evident and stable audiological gain and mid-term functional benefits after the implantation of the device.

### Surgery, healing, state of soft tissue

The mean surgery duration in our study was 64.5 min and was within the range of previously published durations of 40 to 87.2 min [5, 6, 27, 28].

The surgical guide provided by the manufacturer suggests the following surgical approaches: post-auricular incision with superior extension, inferior postauricular incision with extension and posterior C-shape incision [26]. However, as these recommended incisions were often insufficient, many modifications have also been described in the literature. A team from Sheffield Teaching Hospitals adopted a versatile ‘Sheffield-S’ post-auricular incision across the waist of the implant, which remains hidden in the hairline and, according to the authors, optimises the surgical field and provides tension-free wound closure [27]. Deep et al. described two alternative incisions used by American surgeons, invented to avoid placing an incision at the wound tension site: a linear incision placed horizontally over the intended site of the implant screw and an inverted U-shaped incision that passes through the “waist” of the device [29]. A similar incision over the piezoelectric transducer (horizontal or curvilinear) has also been described by Hicks et al. [30]. In children, a minimally invasive approach to Osia implantation (MOSIA) with a transverse skin incision over the implant and the use of an endoscope was described [31]. In our cases, we used a modified inferior postauricular incision. It was a C-shaped incision anterior and inferior to the device. Such an incision was at the suggested distance from the device (> 15 mm) and enabled us to have good bone exposure and comfortably drill a hole for the BI300 implant and polish the bone if required.

As the thickness of the soft tissue in the coil region in our group was always less than 9 mm, no soft tissue reduction was needed in any case. Instead, bone polishing was performed in 14 (70%) cases, but was significant in 6 (30%). Other authors also describe no need for soft tissue reduction [5, 6] or reduction in only a few cases [27], but bone polishing was performed in 41–53% of cases [5, 6, 27].

Except for two cases with minor hematoma and two other cases with temporary pain, we reported no intraoperative or postoperative complications and no problems with healing and soft tissue state in the operated area during the one-year follow-up. In the literature, some minor to moderate complications or adverse effects have been described. Goldstein et al. in their evaluation of 44 Osia^®^ 2 surgeries described an exposure of the dura with bleeding from the transverse sinus (despite this, the BI300 implant fixture was successfully placed), two postoperative wound infections (successfully treated with antibiotics) and two postoperative hematomas (one resolved after application of cold compresses and the other was treated by needle aspiration) [6]. Briggs et al. described three moderate events in a group of 29 adults who underwent implantation: pain in one patient and wound infections in two patients, who were successfully treated with antibiotics [5]. Serious skin-related events requiring the device to be explanted or replaced have been reported in the literature with the previous OSI100 device [17, 23, 24], however, Young et al. described one patient with OSI200 with extrusion of the device requiring surgical removal [7].

In our cases, the aesthetic result was also very good, however, in several patients (especially with thin soft tissue) the retroauricular bump was more or less visible. For one bald man with unilateral conductive hearing loss, it was a significant problem. This patient was also not satisfied with the sound quality. Despite many system regulations and even replacement of the sound processor, he became a non-user and the device was eventually explanted at the request of the patient 2 years after implantation. Cowan et al. also reported a case of a non-user who was dissatisfied with the sound quality of the device [25]. Arndt et al. described a modification of the surgery to reduce the retroauricular bump by creating an implant well in the bone behind the ear for the transducer [32]. In our opinion, such a technique is a good idea, especially for bald patients with thick soft tissue and thin bone.

### Audiological benefits

We have performed a series of audiological tests and found a substantial improvement after implantation of the Osia^®^ 2 device in comparison to unaided hearing, both in pure tone audiometry and speech understanding. Clinically relevant improvement was observed immediately after sound processor’s activation (1 month after surgery) and was stable after 6 and 12 months. The mean functional gain one year after surgery was 47.4  dB HL, and the obtained results were 8.6 dB HL better than those for the Baha^®^ 6 Max processor on the Softband. A significant improvement in speech understanding in the free field both in quiet and noise was also observed with the implanted Osia^®^ 2 device in comparison to the unaided situation. For silent, medium and loud speech (50 dB, 65 dB and 80 dB), the mean improvements one year after surgery in comparison to the unaided situation were 59.5%, 46.5% and 13.3% for speech in quiet and 38.5%, 62% and 36.5% for speech in noise. The results of the Polish adaptive sentence test in noise showed a significant improvement in mean SNR, which amounted to 8.78 dB SPL one year after surgery. The results of the bone conduction (BC) in situ measurements with the implanted Osia^®^ 2 device were better in comparison to the preoperative levels with the Baha^®^ 6 Max on the Softband for all analysed frequencies.

Our audiological results are consistent with those of other studies which have shown significant improvement after Osia^®^ 2 implantation. Briggs et al. published the audiological results of 27 adult subjects (23 with mixed hearing loss or conductive hearing loss and 4 with single-sided deafness) who received implants in three centres (Melbourne, Sydney and Hong Kong) and found at 6-month follow-up after surgery a statistically significant and clinically relevant improvement of 28.4 dB in pure tone average compared to the unaided situation [5]. The improvement was particularly prominent at higher frequencies and provided an average gain of 36.1 dB at 6000 Hz compared to the unaided and 13.9 dB compared to the Baha^®^ 5 Power on the Softband. In speech audiometry in noise, a mean improvement in signal-to-noise ratio of 8.8 dB in comparison to the unaided situation was observed. The improvement in the word recognition score in quiet was 62.3% for 50 dB SPL, 54.0% for 65 dB SPL and 24.3% for 80 dB SPL. Mean improvements in speech recognition scores in quiet of 10.7, 8.8 and 4.0% points were also measured with the Osia^®^ 2 device compared to the preoperative situation with the Baha^®^ 5 Power on the Softband at 50, 65 and 80 dB SPL, respectively. Twenty patients from this group (17 with mixed or conductive hearing loss and 3 with single-sided) were followed up between 12 and 24 months post-implantation, and their results were presented in the next study by Cowan et al. [25]. Between 6- and 24-month follow-up, there were no statistically significant changes in free-field hearing thresholds or speech reception thresholds in noise, indicating that the aided improvements were maintained up to 24 months of follow-up. At the 12-month follow-up, the mean improvement in PTA4 was 26.7 dB HL and the mean SRT in noise improved was 8.7 dB SNR from the unaided situation. Young et al. in a retrospective study analysing 30 adult patients with conductive hearing loss, mixed hearing loss or single-sided deafness, described significant improvements in hearing and speech recognition scores after Osia^®^ 2 implantation compared to preoperative unaided hearing in CNC (14% vs. 80%), AzBio in Quiet (26% vs. 94%), and AzBio in Noise (36% vs. 87%) [7]. The average results for patients with mixed and conductive hearing loss in the unaided situation and with Osia^®^ 2 were as followed: CNC (12% vs. 75%), AzBio in Quiet (23% vs. 90%), and AzBio in Noise (37% vs. 88%). A Systematic Review and Meta-Analysis (14 studies, 314 cases) of the Osia^®^ device (Osia^®^ 1 and Osia^®^ 2) showed that the pooled functional gain for all types of hearing loss was 35.0 dB sound pressure level (SPL) and for conductive/mixed hearing loss it was 37.7 dB SPL compared to unaided hearing [33]. Good audiological results after Osia^®^ 2 implantation have also been described in paediatric cases [28, 34]. You et al. in a group of 16 paediatric patients (10.2–17.6 years) with conductive or mixed hearing loss found a mean functional gain of 43.1 dB [28]. Florentine et al. also highlighted excellent audiologic outcomes without high frequency roll-off [34].

### Quality of life

In our group of implanted patients, the number of hearing problems evaluated with the APHAB scale in different acoustical situations was significantly reduced after implantation in global score and all subscales except aversiveness. One year after surgery, the mean improvement in the global score, ease of communication score, background noise score and reverberation score was 31.1, 30.0, 37.1 and 26.3, respectively, in comparison to the unaided situation. Similarly, an evident improvement in speech, spatial and quality of hearing measured by the SSQ scale was observed after the implantation of the Osia^®^ 2 system, which was 4.51, 3.15 and 3.44 for speech, spatial and quality subscales, respectively.

Good quality of life results after the Osia^®^ 2 surgery have been presented in previous studies. Briggs et al. in a group of 27 cases found statistically significant and clinically relevant mean improvements 6 months after surgery for the mean APHAB global score and all APHAB subscale scores, except the aversiveness score, and for all SSQ subdomains: total score, speech score, spatial score, and quality score [5]. The mean improvement for the APHAB global score was 25.9, for the ease of communication score 27.4, for the background noise score 28.7 and for the reverberation score 31.9. The mean improvement for the SSQ total was 2.50, for the SSQ speech 2.68, for the SSQ spatial 2.30 and for SSQ qualities 2.41. The authors also found a clinically relevant and statistically significant improvement in the HUI scale for comprehensive health state and in the hearing domain. Cowan et al. compared the investigational device at the 12- and 24-month follow-up to the aided situation at the last performed assessment at the 6-month follow-up in the previous study and found no statistically significant changes for any of the APHAB subscale scores and any of the SSQ scores, which indicates that the benefits are stable over the time [25]. In another study, Young et al. demonstrated a significant improvement in quality of life using the Glasgow Benefit Inventory (GBI) survey after Osia^®^ 2 implantation, with patients achieving an average increase in heath satisfaction of + 54.1 points [7]. Of the twelve evaluated patients, ten had a positive perceived improvement in their quality of life. Kim et al. also found a significant subjective benefit from the Osia^®^ 2 system compared to their preoperative status as assessed using the APHAB and SSQ questionnaires [35].

### Strengths and limitations of the study

The strengths of our study are: (1) a homogeneous group of patients: adults with conductive or mixed hearing loss (we had no patients with SSD in the group analysed), (2) surgeries performed by a single experienced otosurgeon and (3) at least one-year follow-up. The limitations of the present study are the limited number of cases and the lack of comparison with other bone conduction implants.

## Conclusions

The implantation of the Osia^®^ 2 device is an effective treatment option for patients with unilateral and bilateral, mixed and conductive hearing loss. The surgery is relatively easy and safe. During the 12-month follow-up, there were no significant problems or complications, either postoperatively or caused by the pressure of the magnet. Implantation of Osia^®^ 2 significantly improves speech understanding in noise and reduces communication problems.

## Data Availability

The preliminary data were presented during OSSEO 2023 - the 8th International Congress on Bone Conduction Hearing and Related Technologies, Denver, USA, September 06–09, 2023 and during CI2024 – the 17th International Conference on Cochlear Implants and other Implantable Technologies, Las Palmas de Gran Canaria, Spain, February 21–24, 2024.
